# Dysregulation of the Cannabinoid System in Childhood Epilepsy: From Mechanisms to Therapy

**DOI:** 10.3390/ijms26136234

**Published:** 2025-06-27

**Authors:** Gloria Montebello, Giuseppe Di Giovanni

**Affiliations:** 1 Department of Physiology and Biochemistry, Faculty of Medicine and Surgery, University of Malta, 2080 Msida, Malta; gloria.montebello@gov.mt; 2 School of Biosciences, Cardiff University, Cardiff CF10 3AT, UK; 3 College of Medicine, Korea University, Seoul 02841, Republic of Korea; 4 Department of Medical and Surgical Sciences, University of Magna Graecia, 88100 Catanzaro, Italy

**Keywords:** endocannabinoid, epileptogenesis epilepsy, pediatric, cannabis, medicinal marijuana, psychiatric comorbidities

## Abstract

Epilepsy affects over 12 million children worldwide, with approximately 30% classified as having drug-resistant epilepsy (DRE), often accompanied by neuropsychiatric comorbidities that severely impact quality of life. The endocannabinoid system (ECS) functions as a multifaceted neuromodulatory network regulating neuronal excitability, synaptic plasticity, and immune homeostasis from early life through adolescence and into aging. In pediatric epilepsies, alterations in ECS components, particularly CB1 receptor expression and endocannabinoid levels, reveal disorder-specific vulnerabilities and therapeutic opportunities. Cannabidiol (CBD), a non-psychoactive compound from *Cannabis sativa*, has shown strong preclinical and clinical efficacy in treating DRE and is approved for Dravet syndrome, Lennox–Gastaut syndrome, and Tuberous Sclerosis Complex. Other ECS-based strategies, such as the use of CB1 receptor-positive allosteric modulators, can selectively enhance endogenous cannabinoid signaling where and when it is active, potentially reducing seizures in conditions like Dravet and absence epilepsy. Similarly, FAAH and MAGL inhibitors may help restore ECS tone without directly activating CB1 receptors. Precision targeting of ECS components based on regional expression and syndrome-specific pathophysiology may optimize seizure control and associated comorbidities. Nonetheless, long-term pediatric use must be approached with caution, given the critical role of the ECS in brain development.

## 1. Introduction

Epilepsy is among the most common and disabling neurological conditions [1]. Due to the wide variety of epilepsy types, it is classified as a spectrum disorder [2]. A “seizure” is defined as a paroxysmal alteration in neurological function resulting from excessive, hypersynchronous neuronal discharge in the brain. Specifically, an “epileptic seizure” is caused by abnormal neuronal firing and must be distinguished from non-epileptic events, such as psychogenic seizures [3]. Epilepsy refers to a chronic condition of recurrent, unprovoked seizures caused by an underlying brain dysfunction [4]. In contrast, seizures resulting from reversible insults, such as fever, are classified as secondary and do not constitute epilepsy. The factors disrupting the normal balance between neuronal excitation and inhibition can be either genetic or acquired [1]. “Epilepsy syndrome” is characterized by a cluster of clinical features, including specific seizure types, characteristic EEG findings, triggers, prognosis, and responses to antiseizure medications (ASMs). Approximately 75% of epilepsy cases are first diagnosed in childhood, reflecting the heightened vulnerability of the developing brain to seizures [5]. Due to underlying physiological reasons, the developing brain is especially prone to seizures, but appears to be more “resistant” to the toxic effects of glutamate than the mature brain [6]. Excitatory synaptic function develops before inhibitory synaptic functions, favoring increased excitation and leading to seizure generation [7]. Additionally, GABA neurotransmission “induces” rather than “inhibits” in early life. GABA-releasing synapses are formed before glutamatergic contacts, and only the delayed expression of a chloride exporter leads to a negative shift in the reversal potential for chloride ions [8]. However, compared to those in the adult brain, seizures in the developing brain seem to cause less structural damage [9].

The annual incidence of childhood epilepsy is approximately 35 per 100,000 individuals [10]. Of these, nearly 30% have drug-resistant epilepsy (DRE), meaning their seizures do not respond to conventional ASMs [11]. This pharmacoresistance profoundly impacts the quality of life of affected children and their families, leading to social isolation, physical limitations, and emotional distress [12].

Despite the advances in ASM development, many forms of epilepsy remain challenging to treat, and the side effects of these drugs can further complicate management. In recent years, medicinal cannabis has emerged as a promising alternative therapy for epilepsy, particularly in children with DRE [13].

The use of cannabis as a medical treatment is not a new phenomenon. Historically, cannabis has been used for medicinal purposes since ancient times [14]. However, in recent years, interest in cannabis-based treatments for epilepsy has surged, particularly with the growing body of evidence supporting its efficacy in treating drug-resistant seizures, although the first evidence dates back to the late 1970s [15]. This growing interest is largely due to the repressive laws surrounding cannabis use, including in the research, which have only recently begun to loosen [16]. Cannabinoids, the active compounds in cannabis, interact with the endocannabinoid system (ECS), which plays a key role in regulating neuronal activity and excitability [17]. Research has suggested that cannabinoids may help to reduce seizure frequency and severity by modulating a plethora of mechanisms from neurotransmitter release, inflammation, to ion channel activity in the brain and many others [14].

Recent randomized clinical trials (RCTs) have provided robust evidence supporting the therapeutic efficacy of cannabinoids in the treatment of pediatric epilepsy. On 25 June 2018, the U.S. Food and Drug Administration (FDA) approved the first plant-derived, purified pharmaceutical-grade cannabidiol (CBD) medication, Epidiolex^®^, for patients aged 2 years and older with Dravet syndrome (DS) or Lennox–Gastaut syndrome (LGS) [18]. Epidiolex^®^ has provided reassurance to parents, as it does not cause euphoric effects, and no cases of addiction have been reported. The approval of Epidiolex^®^ represents a significant step forward in the medical use of cannabis-derived products [19].

In Europe, Epidyolex^®^ was approved by the European Commission on 19 September 2019 for adjunctive therapy in patients aged 2 years and older with seizures associated with DS or LGS, when used in conjunction with clobazam [20]. Successively, CBD was approved for treatment of seizures associated with Tuberous Sclerosis Complex (TSC), in patients aged 1 year and older in the USA [21] and at least 2 years of age in Europe [20].

Despite the promising findings, the use of cannabinoids in children with epilepsy remains a topic of controversy and debate. One of the key concerns is the potential long-term effects of cannabinoids use on brain development are not yet fully understood. Therefore, while cannabinoids may offer a valuable option for treatment-resistant epilepsy, further research is necessary to establish optimal treatment protocols, assess long-term safety, and address potential risks.

While many reviews have been written on cannabinoids and epilepsy [14,22,23,24,25,26,27] and on pediatric epilepsy [11,13,28,29,30,31,32], this review will cover new aspects, such as the alterations of the ECS in animal models and humans with pediatric epilepsy. A deeper understanding of cannabinoid mechanisms, particularly through the study of the impairment of the ECS in pediatric epilepsy, is crucial for advancing cannabinoid-based therapies as novel ASMs.

## 2. Pediatric Epilepsy

While a wide range of childhood epilepsies exist, as discussed in the next paragraph, we will focus here on those for which there is the most compelling evidence of involvement of the ECS. Specifically, we will briefly describe the pathophysiological features of febrile infection-related epilepsy syndrome (FIRES), DS, LGS, pediatric temporal lobe epilepsy, and childhood absence epilepsy (CAE). These epileptic syndromes are frequently associated with neuropsychiatric comorbidities, highlighting the importance of integrated and targeted therapeutic strategies [33].

### 2.1. Classification of Pediatric Epilepsy

The recent updates by the International League Against Epilepsy (ILAE) in classifying seizures and epilepsies have significantly impacted the understanding and management of pediatric epilepsy. The new classifications continue to focus on clinical and electroencephalography (EEG) features but introduces several important changes in terminology and structure [16,34]. The updated seizure classification includes four main categories: focal, generalized, unknown, and unclassified [34]. It distinguishes *classifiers*, which reflect biological and clinical significance, from *descriptors*, which detail additional features. Focal and unknown seizures are further categorized based on the patient’s consciousness during the event, assessed clinically. Generalized seizures are divided into absence, generalized tonic–clonic, and other types, now including negative myoclonus. A basic version notes the presence or absence of visible signs, while an expanded version outlines the seizure’s chronological semiology. With 21 defined seizure types, the classification emphasizes global usability, aiming to create a unified language for clinicians, patients, and caregivers across diverse settings [34] (Table 1).

Febrile infection-related epilepsy syndrome (FIRES), which primarily affects children and leads to refractory seizures following a febrile illness, is classified under *unknown* and *immune* causes, depending on the suspected immunological mechanisms [16]. Dravet syndrome, a severe genetic epilepsy syndrome that begins in infancy, is categorized under *genetic causes*, specifically developmental and epileptic encephalopathies. Similarly, Lennox–Gastaut syndrome, which is characterized by multiple seizure types and cognitive impairment, is also a *genetic* and *structural* epilepsy syndrome.

Pediatric temporal lobe epilepsy (TLE) often falls under *structural causes*, where focal onset seizures, typically starting in the temporal lobe, are associated with a variety of brain abnormalities, such as hippocampal sclerosis. Absence seizures, frequently observed in children, are classified as *generalized onset* seizures with a strong genetic basis, commonly linked to genetic syndromes, like childhood absence epilepsy.

The refined ILAE classification system, coupled with advances in genetic and structural diagnostics, facilitates more accurate diagnoses and individualized treatment plans. This approach, outlined in the 2022 update, underscores the importance of a comprehensive, etiology-based understanding of pediatric epilepsy for both the clinicians and researchers [16] (Table 2).

### 2.2. Febrile Infection-Related Epilepsy Syndrome (FIRES)

FIRES is a rare, devastating epileptic encephalopathy affecting previously healthy school-aged children. It manifests as explosive onset status epilepticus [36], followed by chronic refractory epilepsy with cognitive decline [37]. First described in 1986 [38], FIRES is characterized by fever preceding seizures [39], focal seizures involving the perisylvian and frontotemporal regions, and severe cognitive and behavioral impairments [40]. Current treatments, including barbiturates and immunotherapy, show limited efficacy [41].

### 2.3. Dravet and Lennox Gastaut Syndrome

DS is an early-onset treatment-resistant epilepsy. It typically presents during the first year of life with prolonged febrile and afebrile hemiclonic or generalized clonic seizures [42]. The syndrome affects males twice as often as females, causing epileptic encephalopathy. In 70% to 80% of patients, de novo mutations of the *SCN1A* gene encoding Nav1.1 were identified [43].

In children between one and four years of age, additional seizure types develop, including myoclonic and atypical absences, focal seizures, and generalized tonic–clonic seizures. Over time, seizures develop to become less frequent and more severe in adolescence; however, fever sensitivity persists [44].

In both the convulsive and nonconvulsive types, status epilepticus commonly occurs in patients with DS. This state may be life threatening, while its symptoms may be subtle and difficult to identify. The most common adult seizure type is generalized tonic–clonic. This may have a focal onset but it occurs mainly during sleep. Common treatments include valproic acid, clobazam, and a ketogenic diet [45]. LGS is considered to be an epileptic encephalopathy or a second network epilepsy. It is defined by a triad of drug-resistant seizure types. A typical EEG pattern shows bursts of slow spike–wave complexes or generalized paroxysmal fast activity and intellectual disability. The etiology of LGS varies among genetic, structural, or metabolic factors and is due to an unknown cause [46]. LGS is usually drug resistant, and complete seizure control is not possible. Beyond recurrent seizures, DS is associated with a high rate of premature mortality, affecting approximately a fifth of patients [47].

Valproate, lamotrigine, and topiramate are considered first-line therapeutic drugs for reducing the frequency of seizures. Long-term outcomes are usually poor since the syndrome is associated with long-term adverse effects on intellectual development, independent living, and social functioning [48]. Multiple genetic animal models exist for studying DS, which aim to replicate *SCN1A* loss-of-function observed in DS [49]. Homozygous Scn1a knockout mice develop ataxia and die at 15 days postnatal, whereas heterozygous Scn1a-deficient mice show seizure activity and early mortality starting at 3 weeks of age [50,51]. The hybrid heterozygous Scn1a^+^/^−^ mouse is among the most established. Created by crossing 129S heterozygous males with C57BL/6 wild-type females, these mice exhibit hallmark DS features, including spontaneous seizures, early mortality, and behavioral comorbidities, such as social deficits, anxiety, and cognitive impairments [52]. Their consistent phenotype makes them a standard preclinical model for testing therapies targeting both seizures and associated neuropsychiatric symptoms [53,54].

#### Neuropsychiatric Comorbidities

Individuals with this condition often face a wide range of debilitating comorbidities, including delayed psychomotor development, abnormal gait, hyperactivity, attentional difficulties, autism spectrum features, disrupted sleep, anxiety, depression, language delays, and profound cognitive impairments, all of which significantly diminish quality of life [55]. Current therapeutic approaches typically involve polytherapy with ASMs, such as valproate and clobazam [56]. Nevertheless, seizure control remains inadequate for many patients, and these treatments frequently lead to serious neurological and psychiatric side effects, including increased anxiety, depressive symptoms, and cognitive deficits such as memory loss [57].

### 2.4. Pediatric Temporal Lobe Epilepsy

Pediatric temporal lobe epilepsy (TLE) represents a distinct clinical entity, differing significantly from adult-onset TLE in terms of semiology, neurodevelopmental implications, and outcomes [58]. The incidence of pediatric epilepsy ranges between 33 and 82 per 100,000 annually, with TLE accounting for approximately 8% of cases, often associated with focal seizures [10].

In children under six, TLE’s semiology often lacks classical features observed in adults, such as automatisms and aura, complicating localization. Instead, generalized symptoms, like behavioral arrest and ictal motor manifestations, predominate, reflecting the immature central nervous system’s incomplete myelination [59]. Older children (6+ years old) show semiology closer to adults, including dystonic posturing and oroalimentary automatisms, aiding in lateralization [60].

Hippocampal sclerosis, cortical dysplasia, and low-grade tumors are common etiologies of medically intractable TLE. Although antiepileptic drugs (AEDs) remain first-line treatments, intractable cases often require surgical intervention. Early surgical evaluations, leveraging MRI, EEG, and advanced imaging techniques, like PET and SISCOM, are crucial for optimizing outcomes [61]. Post-surgical seizure freedom rates are in the range of 58–91%, with cognitive benefits often observed, especially in younger children [62]. However, neurodevelopmental and psychiatric challenges, including memory impairments and behavioral disorders, frequently accompany pediatric TLE, necessitating comprehensive management approaches [63].

Overall, pediatric TLE requires prompt diagnosis and tailored interventions to mitigate its impact on cognitive development and quality of life [58].

### 2.5. Childhood Absence Epilepsy (CAE)

Poupart in 1705 first described childhood absence epilepsy (CAE) and Esquirol soon after the term “petit mal” was introduced [64]. In 1854, Delasiauve classified absences as a seizure type with lower severity [65]. Childhood absence seizures (ASs) are genetically determined, but the exact mode of inheritance of the involved genes has not been determined [66]. Typical ASs are classified by the ILAE among generalized nonmotor seizures [35]. ASs have a higher prevalence in females compared to males and typically develop in childhood between the ages of 4 and 10 years, although seizures with later onset have also been recorded [67]. Typical ASs persist for 6.6 years, disappearing between the ages of 10.5 and 14 years, with the age of onset and medication efficacy being strong determinants of seizure course, though the tendency for ASs to cease is present at all ages, not just at puberty [68]. ASs on EEG show generalized 2.5–4 Hz spike-and-wave discharges (SWDs), often triggered by hyperventilation and, less commonly, photic stimulation, with brief lapses of consciousness, eye closure, eyelid movements, and oral automatisms [4,66,69]. Depending on the context of epilepsy, typical ASs may occur as the only seizure type or as generalized tonic–clonic seizures or myoclonic seizures. The presence of perioral myoclonia, single violent jerks, multiple spikes, or spikes coexisting with myoclonic jerks during the ictus of an AS indicates a worse prognosis [70]. ASs result from abnormal corticothalamic network activity, leading to synchronous SWDs and transient consciousness impairments [69,71]. While enhanced thalamic tonic inhibition via extrasynaptic GABA_A_ receptors contributes to ASs [72] recent studies highlight the critical role of cortical tonic inhibition [73] and parvalbumin-expressing interneurons [74]. McCafferty et al. [75] demonstrated that cortical and thalamic neurons show rhythmic but decreased firing during ASs.

Typical ASs of idiopathic generalized epilepsies consist of sudden, brief periods of a loss of consciousness, which are accompanied by synchronous, generalized spike-and-wave discharges (SWDs) in the EEG [66,69]. Similar ASs are exhibited by diverse genetic rat models, including the Genetic Absence Epilepsy Rats from Strasbourg (GAERS) [76] and Wistar Albino Glaxo/Rij (WAG/Rij) rats [77] and mice models, such as stargazer (STG) [78] and GABAγ2(R43Q) [79]. SWDs originate from abnormal firing in thalamic and cortical networks, and GABA_A_ inhibition is integral to their appearance [66,72]. Moreover, an important AS modulation occurs via the basal ganglia [80], and changes in the firing of GABAergic nigral neurons modulate ASs both indirectly via the nigra/superior colliculus/thalamic projection [80] and directly via the nigro-thalamic pathway [81]. Despite the progress made in advancing our understanding of the neuropathological mechanisms underlying AS [75], there are still many unanswered questions, including the mechanism of action of the gold-standard anti-absence drugs, such as ethosuximide (ETX), valproate, and lamotrigine. Failure of monotherapy with gold-standard anti-absence drugs in >50% of childhood/juvenile absence epilepsy [82], the high neuropsychiatric comorbidity rate even after seizure control [83], and the risk of developing generalized seizures [84] increase the demand novel therapeutic approaches.

#### Neuropsychiatric Comorbidities

ASs in children and teenagers were in the past considered relatively benign because of their nonconvulsive nature and high remittance rate in early adulthood. However, recent studies on large cohorts of drug-naïve CAE patients have now conclusively demonstrated that 30% of children with AS are pharmacoresistant [82,85], and 60% suffer from various neuropsychiatric comorbidities. These include anxiety, depression, attention-deficit/hyperactivity disorder (ADHD), and learning deficits that may precede epilepsy diagnosis, persist, and even be aggravated after full pharmacological control of the seizures (see [69] and references within).

For instance, 61% of children with CAE had a psychiatric diagnosis, with 43% experiencing linguistic difficulties and 25% exhibiting subtle cognitive deficits. The severity of these comorbidities was associated with factors such as epilepsy duration, seizure frequency, and antiepileptic drug treatment. However, only 23% of the affected children were receiving interventions for these issues [86]. These findings underscore the importance of the early identification and management of neuropsychiatric comorbidities in children with CAE, highlighting the need for holistic treatments that address both seizures and associated behavioral and cognitive impairments [69].

## 3. Cannabinoids

Cannabinoids are bioactive compounds that interact with the ECS, influencing various physiological processes. They are classified into three main types: phytocannabinoids, which are naturally derived from cannabis plants, such as Δ9-tetrahydrocannabinol (∆9-THC) and cannabidiol (CBD) [87]; synthetic cannabinoids, which are artificially produced to mimic or enhance cannabinoid effects, including nabilone and JWH-018 [88]; and endocannabinoids (eCBs), which are endogenous lipid-based neurotransmitters synthesized within the body, such as anandamide (AEA) and 2-arachidonoylglycerol (2-AG) [89]. These compounds play crucial roles in pain modulation, appetite regulation, and neuroprotection.

### 3.1. Phytocannabinoids

Over 120 natural cannabinoids have been found in *Cannabis sativa*. Seven of these compounds are classified as CBD-like compounds, including cannabidiol [90]. The two most relevant therapeutic and abundant components of the plant are ∆9-THC and CBD [91]. Cannabinoids share a heterocyclic terpene–phenolic structure. They readily cross the brain barrier and are very lipophilic. Hence, cannabinoids are distributed to lipid-based tissues, including neuronal cell membranes and brain parenchyma. Because lipids can be stored in lipid-rich tissues for weeks, they may be gradually released into the bloodstream over time [92]. Among non-psychoactive phytocannabinoids, the most well-known is CBD, which has demonstrated therapeutic potential for epilepsy, anxiety, and inflammation [93]. Other notable non-psychoactive cannabinoids include cannabigerol (CBG), which exhibits neuroprotective and anti-inflammatory properties; cannabichromene (CBC), known for its analgesic and anti-depressant effects; and cannabidiolic acid (CBDA), a precursor to CBD with potential anti-nausea benefits. Other cannabinoids with therapeutic potentials include cannabichromenic acid (CBCA) and cannabichromevarinic acid (CBCVA), as well as cannabinoid-like compounds from non-cannabis plants [94,95]. Unlike ∆9-THC, these compounds exhibit a low affinity for CB1 receptors, reducing their psychoactive potential while retaining their medicinal properties [96].

### 3.2. Cannabidiol (CBD)

Growing evidence highlights the multitarget effects of CBD in the human body. CBD modulates the ECS through both direct and indirect CB1R interactions. Although it has low affinity for CB1R, it antagonizes Δ9-THC and AEA, enhances eCB tone by inhibiting AEA breakdown and transport, and increases 2-AG levels. It may also act as a negative allosteric modulator (PAN) at a distinct CB1R site.

**Figure 1:**
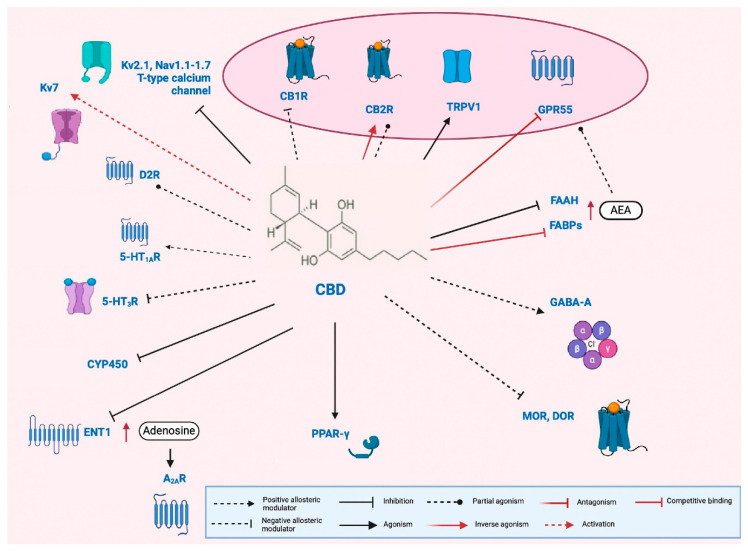
**Pleiotropic mechanism of cannabidiol (CBD).** Cannabidiol (CBD) acts as a negative allosteric modulator at type 1 cannabinoid receptors (CB1Rs) [104], as well as at μ- and δ-opioid receptors (MOR and DOR, respectively) [105], and serotonin 5-HT_3_ receptors (5-HT_3_R) [106]. At type 2 cannabinoid receptors (CB2Rs), CBD has been reported to function both as a partial agonist [104] and an inverse agonist [107]. Additionally, CBD serves as an agonist at transient receptor potential vanilloid 1 (TRPV1) channels [108] and peroxisome proliferator-activated receptor gamma (PPAR-γ) [109], and as a partial agonist at dopamine D_2_ receptors (D2Rs) [110]. It also acts as a positive allosteric modulator at serotonin 5-HT_1_A receptors (5-HT_1_ARs) [111] and GABAA receptors [112]. Furthermore, CBD antagonizes the G-protein-coupled receptor 55 (GPR55) [113]. It may elevate anandamide (AEA) levels by inhibiting fatty acid amide hydrolase (FAAH) [108] and competing with AEA for binding to fatty acid binding proteins (FABPs) [114], thereby indirectly enhancing the activation of CB1R, CB2R, TRPV1, and GPR55. CBD also inhibits the equilibrative nucleoside transporter 1 (ENT1) [115], leading to increased extracellular adenosine and subsequent indirect activation of adenosine A2A receptors (A2ARs). In addition to its receptor-level actions, CBD modulates ion channels by activating voltage-gated potassium channel Kv7 [116], while inhibiting Kv2.1, several voltage-gated sodium channels (Nav1.1 to Nav1.7), and neuronal T-type calcium channels [117]. Finally, in hepatic metabolism, CBD inhibits multiple cytochrome P450 (CYP450) isoforms, raising the potential for drug–drug interactions [118]. Figure modified from [93].

CBD also affects non-CB pathways, modulating inflammation, cell proliferation, and various ion channels (5-HT_1A_R, adenosine A_1_R, CaV, PPARγ, GABA_A_R, and TRP channels). Recent studies have identified potential targets that may explain CBD’s effects on nervous system hyperexcitability. However, further pharmacological research is needed to pinpoint the precise targets and underlying mechanisms [23,97,98,99,100,101,102,103]. Given the ECS’s role in brain development, CBD may produce beneficial or harmful effects in infants depending on the dose and context. Its actions on other targets at low concentrations make its full mechanism unclear (Figure 1).

### 3.3. Tetrahydrocannabinol (∆9-THC)

∆9-THC has two chiral centers in a tricyclic 21-carbon structure in the trans-configuration [90,91]. ∆9-THC has low bioavailability (6–10%), which may be variable due to extensive first-pass metabolism and gastric degradation [119].

Through the oxidation of ∆9-THC, the active metabolite 11-hydroxy-THC is formed by cytochrome P450, 2C9, 2C19, and 3A4 [90]. 11-OH-D9-THC is further oxidized to the inactive THC COOH [120]. THC acts as a partial agonist of CB1Rs in the central nervous system (CNS) [121] and CB2Rs in the immune system [122]. Hence, THC works similarly to eCBs naturally produced by the brain. It has psychotropic effects that involve behavior, cognitive ability, and stability. This effect may be avoided by pretreatment with a recombinant CB1R antagonist [123].

### 3.4. Endocannabinoids

The targets of ∆9-THC and CBD include the ECS but also other systems [123]. The ECS includes two main cannabinoid receptors, CB1/CB2 [124], and endogenous ligands, eCBs, and the enzymes responsible for their synthesis and degradation [123]. The ECS is elaborate and complex; CBRs and other targets respond to endogenous eCBs and to exogenous substances produced by the cannabis plant [125]. The ECS may be activated by three types of ligands. These can be either eCBs produced within the organism or phytocannabinoids produced by the cannabis plant or synthetic cannabinoids if synthesized in the laboratory [126]. 2-arachidonoylglycerol (2-AG) [127] and N-arachidonolethanolamide (AEA, anandamide) [128] are both endogenous ligands that activate CB1R and CB2R. 2-AG is found centrally and peripherally in the hippocampus, brainstem, striatum, and medulla. AEA is found at high concentrations in the brain, mainly in the striatum, hippocampus, and brainstem, and to a lesser extent in the cerebral cortex and cerebellum. To synthesize 2-AG, the enzyme diacylglycerol lipase (DAGL) must catalyze a reaction involving diacylglycerol. Alternatively, 2-AG can be broken down by the substance fatty acid amide hydrolase (FAAH) or by monoacylglycerol lipase (MAGL) into arachidonic acid and glycerol. The synthesis of AEA requires a hydrolysis reaction and phospholipase D [126].

## 4. The Endocannabinoid System in Relation to Pediatric Epilepsy

**Figure 2:**
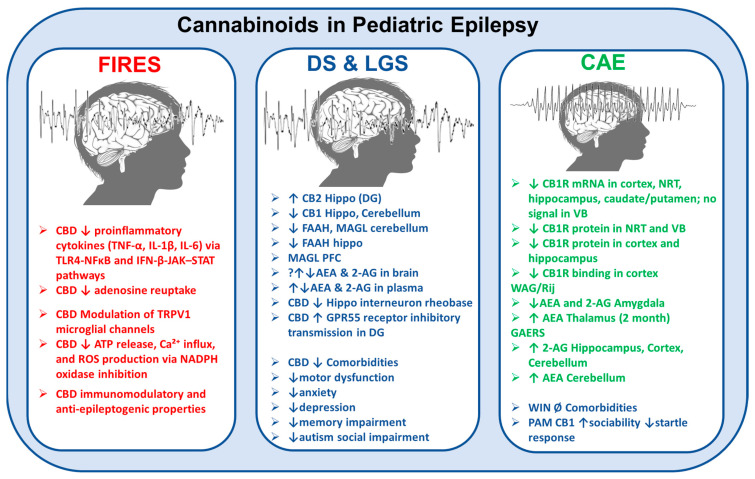
**Cannabinoid system alterations in and effects on pediatric epilepsy syndromes.** This schematic illustrates the pathophysiological changes in the endocannabinoid system (ECS) and the modulatory actions of cannabidiol (CBD) and other cannabinoids across three major pediatric epilepsy syndromes: febrile infection-related epilepsy syndrome (FIRES), Dravet syndrome (DS), and Lennox–Gastaut syndrome (LGS), and childhood absence epilepsy (CAE). FIRES (left panel): CBD reduces the release of proinflammatory cytokines (e.g., TNF-α, IL-1β, IL-6) via TLR4–NFκB and IFN-β–JAK–STAT signaling pathways, inhibits adenosine reuptake, modulates TRPV1 channels on microglia, and suppresses ATP release, Ca^2+^ influx, and reactive oxygen species (ROS) production through NADPH oxidase inhibition. These mechanisms underlie CBD’s anti-inflammatory, immunomodulatory, and antiepileptogenic properties. DS and LGS (center panel): Observed alterations include the upregulation of CB2 receptors in the hippocampus and CB1 receptors in the hippocampus and cerebellum, along with a decreased expression of FAAH and MAGL enzymes in various brain regions. CBD reduces hippocampal interneuron rheobase and enhances inhibitory transmission via GPR55 receptors in the dentate gyrus. Clinically, CBD ameliorates associated comorbidities, such as motor dysfunction, anxiety, depression, memory impairment, and autism-related social deficits. CAE (right panel): There is a significant downregulation of CB1R mRNA and protein in the cortex, hippocampus, and thalamic regions (nucleus reticularis thalami [NRT] and ventrobasal complex [VB]). In Wistar Albino Glaxo/Rijswijk (WAG/Rij) rats and Genetic Absence Epilepsy Rats from Strasbourg (GAERS), two well-established rodent models of CAE, region-specific alterations in endocannabinoid levels have been reported. These include increased anandamide (AEA) concentrations in the thalamus and cerebellum, and elevated levels of 2-arachidonoylglycerol (2-AG) in the hippocampus, cortex, and cerebellum. While the synthetic CB1 receptor agonist WIN 55,212-2 has shown no significant effects on associated neuropsychiatric comorbidities, positive allosteric modulators (PAMs) of CB1 receptors have been demonstrated to enhance sociability and reduce exaggerated startle responses (see main text for references). The symbol ↓ indicates a decrease in expression, concentration, or function; ↑ indicates an increase in expression, concentration, or function; ↔ denotes no significant change or an unclear direction; and Ø represents the absence or lack of effect.

The ECS can modify excitatory and inhibitory synaptic transmission within the nervous system. eCBs are known to be “produced on demand”, meaning they are produced in response to physiological needs [129,130]. Recently, however, it has also been shown that 2-AG is “released on demand” and accumulates within microvesicles [131]. Hence, eCBs mediate the depolarized-induced suppression of excitation (DSE) or inhibition (DSI) [132,133]. The severity of seizure has been shown to correlate with eCB concentrations in the brain [14,134]. Disruption of ECS signaling is believed to contribute to epileptogenesis; however, it remains unclear whether the resulting molecular changes drive proepileptogenic processes, seizures, or reactive adaptations [14] (Figure 2).

### 4.1. The Endocannabinoid System and Cannabinoids in Febrile Infection-Related Epilepsy Syndrome (FIRES)

Evidence for ECS dysfunction in FIRES remains limited. However, CBD has demonstrated efficacy as an adjunctive treatment during both the acute and chronic stages of the disease, acting as an immune modulator and an antiepileptogenic agent [135,136]. Despite these findings, CBD is not currently included in the treatments in the acute phase of FIRES [137], and therefore, it is not recommended as a first-line treatment. A recent case report [138] described a young patient with acute-phase FIRES, refractory to standard treatments, who achieved complete seizure control and successful recovery with a combination of vagal nerve stimulation (VNS) and CBD, suggesting that CBD may serve as a potential adjunctive therapy also to VNS.

The role of the ECS and its receptors in mediating the effects of CBD on FIRES has not yet been elucidated. However, CBD’s modulatory effects on neuroexcitability are linked to its ability to target several key molecules involved in the pathogenesis of FIRES and its outcomes. CBD reduced microglia-mediated neuroinflammation by suppressing proinflammatory cytokines and chemokines, including TNF-α, IL-1β, and IL-6, through the inhibition of the TLR4-NFκB and IFN-β-JAK–STAT pathways [139,140]. In addition, its anti-inflammatory effects are linked to the inhibition of adenosine reuptake, which contributes to decreased neuronal excitability [140,141].

Additionally, CBD activates and subsequently desensitizes microglial TRPV1 channels, thereby mitigating sustained neuroinflammation [140,141]. It also reduces ATP release, intracellular calcium influx, and the production of reactive oxygen species (ROS) through the inhibition of nicotinamide adenine dinucleotide phosphate (NADPH) oxidase activity [140] (Figure 1).

### 4.2. The Endocannabinoid System and Cannabinoids in Dravet Syndrome (DS) and Lennox–Gastaut Syndrome (LGS)

#### 4.2.1. Clinical Evidence of Cannabinoid Use in DS and LGS

CBD medication, Epidiolex^®^, has been approved for patients aged 1 year and older with DS or LGS and TSC, in conjunction with clobazam [18]. CBD approval was based on six randomized controlled trials (RCTs) demonstrating a 40 to 50% reduction in seizure frequency among Epidiolex-treated patients [56,142,143,144,145]. A recent retrospective multicenter chart review in Germany observed a reduction in seizure frequency and sustained treatment retention for up to 12 months across different age groups, among patients with severe, treatment-refractory LGS or DS receiving adjunctive CBD and clobazam simultaneously [146]. Several meta-analyses have been published on CBD use in DRE among the adult [147] and pediatric population [148,149,150]. Adding CBD benefits most children with pharmacoresistant epilepsy. While 20 mg/kg/day offers better seizure control than 10 mg/kg/day, the lower dose remains effective and a viable treatment option [148,149,150]. Common adverse effects of CBD include drowsiness, lethargy, diarrhea, loss of appetite, and weight loss [151,152]. DS is a channelopathy mainly linked to *SCN1A* and *SCN1B* mutations, with additional contributions from potassium and calcium channel gene variants. These disrupt excitatory/inhibitory balance through altered channel function [153]. Thus, the modulation of these channels may underlie CBD’s therapeutic mechanism, although current support comes primarily from preclinical studies [153]. CBD may also exert therapeutic effects in DS and LGS by modulating dysregulated components of the ECS, although ECS alterations in these conditions remain poorly characterized. For instance, Rubio et al. [154] reported an increased expression of CB2Rs in lymphocytes derived from DS patients, potentially indicating a compensatory or pathological upregulation in peripheral immune cells. Notably, no significant changes were detected in plasma levels of key endocannabinoid, such as AEA or 2-AG, suggesting that peripheral eCB levels may not accurately represent central ECS dysfunction in these syndromes [154].

#### 4.2.2. Animal Evidence of Cannabinoid Use in DS and LGS

CBD has been shown to attenuate hyperthermia-induced seizures in *Scn1a+/−* mice, which closely mirrors the clinical features of DS [54,155], and reduce the frequency and severity of spontaneous seizures [54]. CBD’s anticonvulsant effects were initially thought to stem from pharmacokinetic and pharmacodynamic interactions with clobazam [56]. However, the preclinical evidence suggests that CBD’s efficacy goes beyond elevated clobazam levels, likely involving the positive modulation of GABA_A_Rs and multimodal engagement of anticonvulsant pathways in DS [155].

Several other cannabinoids have shown efficacy against thermal-induced seizures in *Scn1a+/−* mice. Treatment with phytocannabinoids, such as CBCA and CBCVA [156] CBGA, CBDVA, and CBGA [157], as well as the cannabinoid-like compound magnolol from non-cannabis plant *Magnolia officinalis* [158], reduced spontaneous seizures and improved survival in the *Scn1a+/−* mouse model of DS.

Impairments in the ECS have been proved in the same *Scn1a+/−* mouse model [159,160]. Anderson et al. (2022) [161] identified *Cnr1*, the gene encoding CB1R, as a genetic modifier of epilepsy in *Scn1a+/−* mice, with a deficiency of eCBs serving the pathological background. Reduced hippocampal CB1R expression was observed in this model, while cortical CB1 levels and brain concentrations of major endocannabinoids (AEA and 2-AG) remained unchanged (Anderson et al., 2022). However, lesser-studied monoacylglycerols, such as 2-linoleoylglycerol (2-LG) and 1-linoleoylglycerol (1-LG), were significantly elevated following hyperthermia-induced seizures in *Scn1a+/−* mice [160]. The delayed timing of sampling (5 min post-seizure) in these studies [160,162] may have limited the detection of transient changes in eCB levels. For instance, in vivo two-photon imaging demonstrated significant increases in 2-AG concentrations in the hippocampal CA1 region contextually to electrically induced seizures, while AEA levels remained unchanged [163]. In support of a deficiency of ECS in DS, the positive allosteric modulation (PAM) of CB1 receptor GAT229 and enhancement of brain 2-AG concentrations using ABX-1431, a MAGL inhibitor, both produced significant anticonvulsant effects against hyperthermia-induced seizures in *Scn1a+/−* mice [161].

A novel DS model involving heterozygous, conditional, knock-in mice with a missense mutation (A1783V) in the *SCN1A* gene, expressed exclusively in CNS neurons (*Syn-Cre/SCN1AWT/A1783V*), revealed elevated CB2R levels in the hippocampus, particularly in the dentate gyrus [164]. This upregulation may represent an endogenous protective response aimed at reducing neuroinflammation in DS. Additionally, this model exhibited other alterations in the ECS, including the downregulation of CB1Rs in the cerebellum and hippocampus, changes that may reflect impaired synaptic function and correlate with the motor and memory deficits observed in these mice. Notably, reductions in eCB-inactivating enzymes were detected in specific brain regions: both MAGL and FAAH in the cerebellum, MAGL alone in the prefrontal cortex, and FAAH alone in the hippocampus. These enzymatic changes suggest region-specific elevations in endocannabinoid levels, particularly 2-AG, given the prominent downregulation of MAGL [164]. These findings underscore the potential of targeting the ECS for next-generation anticonvulsant therapies. However, the therapeutic utility of MAGL inhibition may be constrained by its narrow therapeutic range, as subchronic treatment with high doses of ABX-1431 exacerbated spontaneous seizures. This highlights the need for dose optimization and further investigation into the long-term safety and efficacy of this approach [161].

Animal studies on LGS are instead limited, but a promising model has recently emerged: the *Gabrb3*+/D120N mouse, a heterozygous knock-in for the β3 subunit of the GABA_A_R [165]. Acute administration of magnolol significantly reduced the number and duration of atypical ASs in the *Gabrb3*+/D120N mouse model, consistent with an antiseizure effect [158]. In vitro, magnolol inhibited all T-type calcium channel subtypes, similar to other phytocannabinoids [94], but showed no activation of CB1 or CB2Rs [158].

In conclusion, cannabinoid efficacy in DS extends beyond interactions with clobazam, involving the direct modulation of GABA_A_Rs and multiple anticonvulsant mechanisms. Additionally, PAM and elevating eCBs show antiseizure effects through CB1 and CB2R modulation and T-type calcium channel inhibition, underscoring the therapeutic potential of targeting the broader endocannabinoid system.

#### 4.2.3. Cannabinoid Use for Neuropsychiatric Comorbidities in DS and LGS

No direct data are available on the involvement of alterations in ECS in epilepsy-associated comorbidities. However, indirect evidence comes from studies investigating the long-term effects (followed for up to 2 years) of CBD treatment in patients with DRE, where CBD improved mood [166,167] without impairing cognition [168]. Furthermore, in *Scn1a*−/− and *Scn1a*+/− mouse models of DS, CBD administered at anticonvulsant doses improved their welfare by reducing pain and enhancing general health [54,169]. In heterozygous mice, it also alleviated several complex behavioral comorbidities, including motor dysfunction, anxiety-like behavior (assessed in the elevated plus maze), and depression-like behavior (evaluated with the sucrose preference test) [169]. Furthermore, CBD improved spatial learning and memory in the eight-arm radial maze [169] as well as deficits in autism-like social interactions [54,169].

CBD exerts dual actions in the hippocampus of DS mice, contributing to the restoration of excitatory–inhibitory balance. First, it lowers the rheobase of interneurons, thereby enhancing their excitability and addressing deficits in GABAergic firing [50,170]. Second, CBD acts through the GPR55 receptor to enhance inhibitory synaptic transmission onto dentate gyrus granule cells (DGCs), leading to a reduction in their spontaneous firing. Additionally, CBD directly reduces DGC excitability, which may underlie its capacity to decrease both seizure frequency and severity [54].

These findings underscore the complex role of the ECS system in DS and LGS, highlighting its potential as a therapeutic target. With a favorable side-effect profile, emerging evidence supports CBD’s efficacy in treatment-resistant epilepsies and their associated comorbidities. However, further research is required to better understand the underlying mechanisms and the broader therapeutic implications (Figure 1).

### 4.3. The Endocannabinoid System in Other Refractory Pediatric Epilepsies

Several developmental and encephalopathies beyond DS and LGS exhibit DRE, including TSC, Infantile Spasms and Epileptic Spasms, CDKL5 Deficiency and Aicardi, Doose Syndrome, Dup15q Syndrome, and Sturge–Weber Syndrome [171]. These conditions have been treated with pharmaceutical-grade CBD, with reports of reduced seizure severity [171]. A recent systematic review of preliminary open-label studies [172] indicated that purified CBD may be effective in managing otherwise refractory childhood epilepsies. Among these, the most robust evidence supports CBD’s efficacy in TSC, where it has demonstrated seizure reduction as both monotherapy [173] and as adjunctive therapy with clobazam [174], leading to its FDA approval for use in TSC, alongside DS and LGS. However, conventional RCTs often face limitations in rare epilepsy syndromes due to low patient numbers and heterogeneity. As such, novel and adaptive clinical trial designs should be considered to rigorously evaluate the efficacy and safety of CBD in these intractable pediatric epilepsies [175].

### 4.4. The Endocannabinoid System and Cannabinoids in Pediatric Temporal Lobe Epilepsy

Research specifically focusing on the ECS in pediatric TLE remains limited, with no direct data currently available in the pediatric population. Despite this, studies on TLE animal models offer valuable insights into the potential role of the ECS in seizure modulation and the pathophysiology of TLE, which may inform future research in pediatric cases [14]. The effects of CBD have not been investigated in patients with TLE yet, although in vitro evidence from human samples suggests that high concentrations of CBD interacts with 5-HT_1A_ receptors, acting as an inverse agonist [176]. Recent findings show altered receptor-expression hippocampal and cortical microvasculature, CB1, and CB2Rs may offer a potential therapeutic target to preserve blood–brain barrier integrity in drug-resistant mesial temporal lobe epilepsy (DR-MTLE) patients [177]. In DR-MTLE patients, CB1R-induced G-protein signaling microvasculature is increased, along with higher levels of ANA, and reduced levels of 2-AG. These changes are more pronounced in patients without mood disorders, suggesting that altered eCB levels in the hippocampus and temporal neocortex may contribute to MTLE pathophysiology. This supports the idea that increased eCB activity could explain the absence of mood disorders in some MTLE patients, highlighting the complex interplay between the ECS and neurological and psychological aspects [178].

On the other hand, drug-naive TLE patients show increased glutamatergic activity [179] and reduced AEA levels in the cerebrospinal fluid (CSF), while 2-AG levels remain unchanged [180]. Reduced CB1R mRNA and protein expression have been detected in the hippocampus of pharmacoresistant TLE patients [181]. However, PET imaging reveals increased CB1R availability in the temporal lobe ipsilateral to the epileptic focus in MTLE patients [182], suggesting enhanced CB1R binding despite low mRNA and protein expression. This indicates increased CB1R-induced neurotransmission with potential inhibitory effects in epilepsy, though severe hippocampal damage in pharmacoresistant MTLE suggests hypoactive eCB signaling [183]. Hypoactivity of the ECS is linked to anxiety and depression [184], and as pharmacoresistant MTLE often coexists with these conditions [185], hypoactivity may contribute to their comorbidity, though direct evidence is lacking.

Another comorbidity of MTLE is memory impairment [186]; the modulation of hippocampal synaptic plasticity through ECS targeting provides additional therapeutic insights. Notably, the inhibition of FAAH by URB597 suppresses maximal dentate after-discharges [187], restores seizure-induced impairments in short- and long-term plasticity, and avoids the detrimental memory effects associated with synthetic cannabinoid receptor agonists [187]. Moreover, AEA signaling augmentation restores the phasic control of eCB signaling over GABAergic activity and plasticity in the basolateral amygdala (BLA), mitigating seizure-induced alterations in fear memory [188]. This evidence supports the selective enhancement of eCB tone over broad CB1R activation as a promising approach for managing seizures and cognitive impairments in epilepsy. Moreover, interactions between cannabinoid and serotonin systems, as evidenced in TLE models, reveal synergistic neuroprotective effects, further advancing our understanding of their potential [189,190] (Colangeli et al., 2019). Together, these findings highlight the intricate interplay of cannabinoids, eCBs, and serotonin in shaping neuronal excitability and therapeutic strategies for epilepsy and related disorders [184].

### 4.5. The Endocannabinoid System in Childhood Absence Epilepsy (CAE)

#### 4.5.1. Clinical Evidence of Cannabinoid Use in CAE

As far as CAE is concerned, the evidence regarding the involvement of the ECS in human ASs remains conflicting. However, a recent prospective pilot study [191] demonstrated the effect of pharmaceutical-grade CBD on a small cohort of 14 patients with typical CAE. CBD treatment (8–20 mg/kg/day) administered over 90 days appeared ineffective for typical absence seizures and may have even exacerbate them, as several patients exhibited a clinically significant increase in SWDs. Despite this, CBD was generally well tolerated, with only mild side effects reported. However, an anti-absence effect of CBD was observed in 5 out of the 14 (35%) patients enrolled, with 3 patients receiving CBD in combination with ETX and 2 patients on CBD monotherapy [191]. This anti-absence effect was also reported in the pooled analysis of the CBD Expanded Access Program (EAP), which provided treatment to 892 patients with DRE for up to 33 months [192], as well as in 4 out of 5 patients enrolled in a separate EAP conducted in Massachusetts involving 50 patients treated for up to 60 months [193]. However, definitive conclusions from these EAP studies cannot be drawn, as they did not distinguish between typical and atypical CAE, and changes in seizure frequency were not confirmed by EEG monitoring [192].

Two clinical trials (ClinicalTrials.gov Identifiers: NCT03355300 and NCT03336242) were initiated in 2017 to evaluate the antiepileptic efficacy of a CBD oral solution in pediatric patients with treatment-resistant childhood absence seizures. However, both trials were terminated before completion, and no conclusive results were obtained [194]. From the limited information available, it appears that an effect of CBD was observed only at low concentrations (approximately 20 mg/kg/day) in the small cohort of patients enrolled [194]. No studies have specifically investigated the effect of ∆9-THC on absence seizures (ASs), except for a small prospective study on childhood epilepsy that included three patients with idiopathic generalized epilepsy treated with oral cannabis extracts containing both CBD and ∆9-THC [32]. The study yielded inconclusive results regarding whether the combination of ∆9-THC and CBD offers better seizure control, or at what ratio, compared to CBD alone, but it highlighted that the risk–benefit profile may be less favorable than that of pharmaceutical-grade CBD [32]. The major outcomes are summarized in Table 3.

#### 4.5.2. Animal Evidence of Cannabinoid Use in CAE

Although the experimental evidence remains inconsistent, more data are available on the role of cannabinoids in ASs from animal experimental models of the disease compared to other pediatric epilepsies (Table 3). One of the earliest investigations on ∆^9^-THC and CBD SWDs photically evoked showed a lack of effect by CBD and conversely pro-absence activity by ∆^9^-THC [195]. Of note, by analyzing cannabinoids in other animal models of epilepsy, it was proposed that cannabinoids were useful against convulsive seizures but not CAE [195]. For the last few decades, the cannabis research has been hindered by law restrictions and its criminalization in the USA Controlled Substances Act of 1970 [204] determining that cannabis therapeutic potentials were forgotten until recently. In 2010, a study on epileptic WAG/Rij rats [198] showed a complex biphasic modulation of SWDs by a synthetic CB1/2R agonist, WIN 55,212-2 [124]. The authors showed that the acute general administration of high doses (3–12 mg/kg, s.c.) of cannabinoids biphasically modulated SWDs. The doses of 6 and 12 mg/kg decreased the incidence of SWDs in the first 2 and 3 h, respectively, but produced an elongation of the seizures after 3 h, with single SWDs reaching up to 100 s or more at the 6 h mark. The CB1R antagonist/inverse agonist AM251 was ineffective at 6 and 12 mg/kg, but the latter dose blocked the early inhibition in SWDs, the late increase in mean SWD duration, and produced at the 4th and 5th hours an increase in the incidence of SWDs. These data are puzzling, especially since incredibly high doses of cannabinoids were used. WIN 55,212-2 loses affinity for CB1Rs at high doses, and an inhibition of motor activity was shown, which might have caused the early decrease in SWDs. A 12 mg/kg dose of WIN 55,212-2 completely suppressed SWDs in a WAG/Rij rat that developed generalized convulsive seizures after cannabinoid treatment [205], suggesting that CB1R activation may facilitate the transition from nonconvulsive to generalized convulsive seizures.

Nevertheless, more recent investigations did not replicate the anti-absence effect of WIN 55,212-2 [199,205]. Indeed, both acute and subchronic administrations of 6 mg/kg WIN 55,212-2 did not modify the incidence of SWDs, but similarly to the previous study [198] elongated the duration of the SWDs, likely by inhibiting NRT neurons known to be involved in the stopping mechanism of SWDs [206].

Sysoeva et al. (2023) explored the effect of cannabinoids on absence epileptic networks, revealing that WIN55,212-2 modulated the network strength in the frontal and hippocampal areas. It decreased SWD frequency but increased its duration, indicating a complex interaction with epileptic networks and neuronal coupling [205].

Recently, Howland and colleagues reported a contrasting effect of agonist PAMs (ago-PAMs) for CB1Rs [201,203] and the phytocannabinoids ∆^9^-THC and CBD [196] in GAERS. Systemic administrations of ago-PAMs GAT229 and GAT211 reduced SWD incidence and duration by 50% and 40%, respectively [201], while the more powerful ago-PAM GAT591 and GAT593 only decreased the total time spent in seizures by 36% and 34%, respectively, without affecting SWD incidence, average duration, or oscillatory frequency [203]. The anti-absence effects of CB1R PAMs appear CB1R-dependent, as SR141716A, a CB1R antagonist/inverse agonist, blocked GAT211’s effect [201]. In contrast, purified ∆^9^-THC (3–10 mg/kg) exerted a pro-absence effect on male and female GAERS, increasing SWD incidence, duration, and total seizure time, while reducing SWD frequency [196]. Conversely, purified CBD (30–100 mg/kg) did not alter SWD incidence, but reduced seizure duration by shortening the average SWD length. Inhalation of smoke from a high-∆9-THC cannabis strain increased the total and average SWD duration, while leaving the frequency unchanged. These effects were likely not CB1R-dependent, as they were not blocked by SR141716A. On the other hand, exposure to smoke from a CBD-rich cannabis strain had no effect on any SWDs, likely because the CBD plasma concentrations did not reach effective levels [196] (Table 3).

#### 4.5.3. Cannabinoids Infusion in Brain Areas in CAE Animal Models

In WAG/Rij rats, both intracerebroventricular (i.c.v.) and intraperitoneal (i.p.) administrations of AEA and its analog N-palmitoylethanolamine (PEA) reduced SWD incidence and duration. PEA’s effects were blocked by pre-treatment with SR141716 and the PPAR-α antagonist GW6471, whereas AEA’s i.c.v. anti-absence effects were blocked only by SR141716A [202]. Further studies showed that AEA and the CB1R agonist WIN55,212-2, when injected into the thalamic nuclei (NRT, VB), the somatosensory cortex (S1po), or administered i.c.v., also suppressed SWDs. Interestingly, SR141716A alone did not induce a significant pro-absence effect [200].

In GAERS, the infusion of the ago-PAM GAT229 into the motor cortex, a region not directly involved in SWD generation, reduced SWD incidence, average duration, and total duration; this effect was prevented by systemic SR141716A, which alone had no effect [201] (Table 3).

#### 4.5.4. Cannabinoid Concentration in Brain Areas in CAE Animal Models

eCB concentrations in specific brain regions were assessed in 2- and 6-month-old WAG/Rij rats, with ACE rats and normal Wistars as controls [202]. In 6-month-old WAG/Rij rats, the amygdala showed reduced levels of AEA, 2-AG, and PEA, while the cortex exhibited higher levels of PEA, and no significant changes were detected in the thalamus compared to ACE rats [202]. In contrast, adult, symptomatic GAERS exhibited increased 2-AG levels in the cortex, hippocampus, and cerebellum, but not in the thalamus, and elevated AEA only in the cerebellum [201].

#### 4.5.5. Cannabinoid Receptor Expression in CAE Animal Models

In situ hybridization in age-matched WAG/Rij and ACI rats revealed reduced CB1R mRNA expression in the cortex, NRT, hippocampus, and caudate/putamen of WAG/Rij rats, with no detectable signal in the VB [198]. However, Western blot (WB) analysis indicated decreased CB1R protein levels only in the NRT and VB [198]. In GAERS, CB1R expression was examined in the cortex, thalamus, hippocampus, and cerebellum, showing reduced protein levels in the cortex and hippocampus [201]. This cortical downregulation was further confirmed by [^3^H]SR141716A binding analysis via liquid scintillation spectrometry [201].

#### 4.5.6. Modulators of Cannabinoid Synthesis and Breakdown in CAE Animal Models

The two major eCBs, AEA and 2-AG, are both synthesized postsynaptically but act on presynaptic CB1Rs to inhibit neurotransmitter release [207]. Given the adverse effects observed with direct-acting CB1R agonists and antagonists in preclinical rodent models of seizures, several studies have investigated whether enhancing endogenous cannabinoid tone might reduce seizure occurrence and severity [14]. This type of pharmacological intervention has not yet been investigated in CAE; however, our preliminary studies using PF-04457845, a novel FAAH inhibitor [208], have shown a promising anti-absence effect in GAERS (unpublished observations).

#### 4.5.7. Cannabinoids and Neuropsychiatric Comorbidities in CAE Animal Models

Alterations in the ECS have been implicated in anxiety and mood disorders, and the modulation of ECS components may provide therapeutic benefits [184]. Therefore, the neuropsychiatric comorbidities observed in CAE [69] may be responsive to cannabinoid modulators. Nevertheless, studies on GAERS may have limited the ability to provide further insights, as recent evidence shows that NEC rats exhibit higher anxiety-like behavior and neophobia compared to GAERS [209,210,211], contrary to previous assumptions [69]. Additionally, the effects of cannabinoid receptor agonists, such as WIN 55,212-2, appear to be strain-dependent: this compound reduces anxiety in NEC rats but not in GAERS, where it instead produces sedative effects [209]. These behavioral outcomes were paralleled by monoaminergic alterations. GAERS exhibited lower baseline 5-HT levels in the hippocampus and substantia nigra, as well as reduced NA levels in the entopeduncular nucleus, potentially contributing to their hypermotility and reduced anxiety phenotype. Moreover, ECS activation modulated monoamine levels, further implicating ECS involvement in the observed behavioral differences [209]. However, other evidence suggests that the effect of cannabinoid treatment may not be uniform and could be dependent on the behavioral test used. For example, WIN 55,212-2 induced hyperlocomotion in GAERS and did not improve anxiety in the elevated plus maze in NEC rats and GAERS [212] both of which exhibited comparable levels of anxiety-like behavior [212,213]. Consistently, Roebuck et al. [213] also found that CB1R PAM GAT211, while ineffective on anxiety-like behavior in elevated plus-maze and open-field tests or locomotory activity, showed increase sociability and reduced the elevated startle response, particularly in female GAERS [213].

Together, these findings suggest that the GAERS model does not fully replicate the anxiety observed in CAE, and cannabinoids do not significantly alter the emotional state of GAERS. This limits the ability to draw definitive conclusions about the efficacy of cannabinoids in treating comorbid anxiety in children with AS. While the anxiolytic effects of cannabinoids have been consistently observed in normal rats [184], the apparent unresponsiveness in GAERS may be due to impairments in ECS signaling, complicating interpretation. However, the observed effects on sociability and startle response in female GAERS [213] are intriguing, suggesting a potential therapeutic role for cannabinoids in addressing other neuropsychiatric comorbidities. These findings highlight the importance of investigating sex differences in ECS modulation. Further studies are needed to clarify the mechanisms and therapeutic windows of ECS-targeted interventions for both seizures and associated psychiatric symptoms. Critically, the development or selection of more appropriate animal models that better reflect both the seizure phenotype and comorbidities is essential.

Comparing GAERS to WAG/Rij rats reveals a different form of cannabinoid control of SWDs [198,201,205], both in terms of their generation and termination, further increasing the number of differences already described between these two epileptic strains [214].

**Figure 3:**
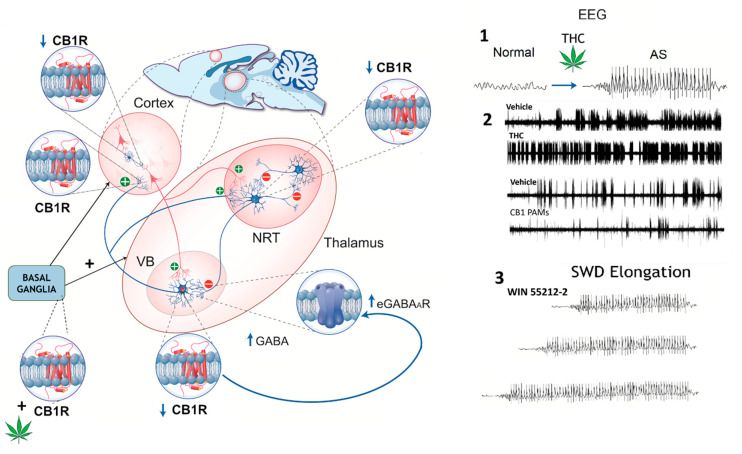
Schematic representation of cannabinoid receptor type 1 (CB1R) distribution in the rat thalamocortical circuit, illustrating region-specific changes in expression (↑ or ↓) and their impact on GABAergic signaling. CB1R expression is decreased in the cortex, thalamic relay neurons (VBs), basal ganglia, and key inhibitory synapses. In the thalamic reticular nucleus (NRT), reduced CB1R expression may contribute to thalamocortical hyperexcitability. Enhanced GABA release in the VB could increase extrasynaptic GABA_A receptor (eGABA_A_R)-mediated tonic inhibition, facilitating the generation of SWDs. Right panels—EEG recordings: 1. Phytocannabinoids (∆9-THC): THC-induced SWDs are hypothesized to result from CB1R activation at striatonigral synapses, which enhances GABA release and disinhibits oscillation-promoting neurons in the nigro-thalamic pathway, triggering SWDs even in previously non-epileptic animals. 2. In GAERS, ∆9-THC exacerbates SWDs by increasing their frequency and duration, while CB1R-positive allosteric modulators (PAMs) reduce SWD incidence. Cannabidiol (CBD) has been reported to suppress SWDs in animal models, although human studies suggest it may paradoxically increase SWD activity in some cases. 3. In WAG/Rij rats, the synthetic CB1R agonist WIN 55212-2 disrupts the termination (“stop”) mechanism of SWDs, likely via effects on the NRT, resulting in prolonged discharges without altering their incidence (see main text for references). Figure modified with permission from [229].

CB1Rs at GABAergic synapses act as strategically placed control points for the activity-dependent regulation of dynamically changing normal and pathological oscillatory network activity [215]. The proepileptic effects of CB1Rs may also involve other brain regions, such as the output structures of the basal ganglia, particularly the substantia nigra pars reticulata (SNr), as shown in a study on normal mice [197]. CB1R activation induced thalamocortical high-voltage spindles (HVSs) (putative SWDs) by selectively increasing the activity in the nigro-thalamic pathway [197]. This may lead to increased GABA release in the thalamus and enhanced GABA_A_R receptor-mediated tonic inhibition, contributing to ASs [72], highlighting a potential risk of CAE induced by cannabis abuse (Figure 3).

Both NRT and TC VB neurons can synthesize and release 2-AG; moderate DAGLα expression in the NRT and VB of adult Wistar rats was observed [216]. Nevertheless, a synapse target-dependent effect was observed in the thalamus, as DSI occurs only at intra-NRT synapses, not to NRT to VB [217]. This 2-AG-mediated DSI may increase NRT neuronal discharges, reducing burst firing due to membrane depolarization [218], or Ca^2+^ influx during a low-threshold spike (LTS) may elevate intracellular 2-AG via DAGLα, depressing LTS burst spikes itself [219]. Summing up, eCBs modulate synaptic strength at specific inhibitory thalamic pathways, dynamically influencing thalamic–cortical synchrony. NRT neurons inhibit either VB neurons, promoting rhythmic oscillations, or neighboring TRN neurons, limiting synchrony. Therefore, the emergence of ASs may result from reduced eCB signaling in the NRT, supported by a reduction of both CB1R mRNA and protein expression [198] (but not in GAERS, in which no thalamic ECS alterations were detected [201]), which weakens intra-TRN inhibition and enhances thalamic synchrony [217]. The long-lasting increase in long SWDs observed after acute and subchronic WIN administrations in adult WAG/Rij rats is likely driven by CB1R activation and a consequent reduction in GABA availability within the NRT [199]. Another brain region implicated in the ictogenesis of ASs is the peri-oral region of the somatosensory cortex (poS1) [66,80], where the dysregulation of the ECS has been observed. In GAERS, reduced CB1R levels in the cortex likely create a permissive environment for increased excitability and seizure propagation [201], whereas no changes in CB1R mRNA expression were detected in WAG/Rij rats [198]. These ECS alterations may contribute to seizure onset during development and their exacerbation during adolescence [201]. The hippocampus (HPC) shows decreased CB1R protein [201] and mRNA expression levels [198], whereas 2-AG levels are elevated in GAERS compared to the controls [201]. These findings are relevant considering the recent evidence of HPC involvement in CAE and the marked decrease in HPC frontal-cortex unidirectional coupling exclusively during SWDs, which was reversed by WIN55,212-2 treatment that effectively reduced ASs [205]. These changes in the ECS in the HPC may help explain the cognitive and behavioral alterations seen in GAERS [210,211,220,221,222,223] and patients with CAE [69].

The effects of CB1R-dependent activation, whether through agonists or ago-PAMs, or CB1R-independent effects mediated by CBD administration on modulating SWDs remain unclear. The pro-absence effects observed after acute ∆9-THC exposure in GAERS [196], and following both acute and subchronic administrations of WIN55,212-2 in WAG/Rij rats [199] limit the therapeutic potential of CB1R orthosteric agonists. In contrast, CB1R ago-PAMs appear more promising, as they do not lead to the overactivation of CB1Rs or associated pro-absence, such as elongated SWD duration [203], nor do they produce psychotropic effects. Since ago-PAMs require the presence of eCBs to exert their action, they provide an intrinsic ceiling effect that reduces the risk of overstimulation.

Although ago-PAM anti-absence effects are blocked by CB1R antagonism, the eCB may also have mixed effects. These include the modulation of TRPV1 channels, inhibition of L- and T-type Ca^2+^ channels [224,225], or direct activation of extrasynaptic GABA_A_Rs [226,227] by binding to their β_2_ subunit [228]. Synaptic GABA_A_R-mediated inhibition has also been reported [227]. Notably, enhanced tonic GABA_A_R inhibition via the δ-containing extrasynaptic GABA_A_ receptor in VB TC neurons is both necessary and sufficient for the expression of ASs in different rodent models, while phasic GABA_A_R inhibition remains unaltered [72]. These observations support the hypothesis of a potential hyper-eCB signal contribution to the pathophysiology of ASs and suggest limitations in the use of cannabinoids, given their contrasting effects on GABA_A_Rs and other targets, for this type of childhood epilepsy. However, further research is needed to explore ECS involvement in both seizures and neurobehavioral comorbidities in CAE (Figure 2 and Figure 3).

## 5. Limitations, Risks, and Future Directions for the Research and Treatment of Cannabinoid Use in Pediatric Epilepsy

The ECS functions as a multifaceted neuromodulatory network that regulates neuronal excitability, synaptic plasticity, and immune homeostasis [207], from early life through adolescence and into aging [123]. Therefore, is not surprising that in various pediatric epilepsies, alterations in ECS components, particularly CB1R expression and brain eCB content, highlight both disorder-specific vulnerabilities and therapeutic opportunities [230].

In the developing brain, the ECS plays a critical role in regulating neural progenitor cell survival, proliferation, differentiation, and migration primarily through CB1 receptor signaling [231]. This system helps shape brain development by modulating synaptic plasticity and maintaining the balance between excitation and inhibition, which is essential for normal cortical formation [232]. ECS activity influences key developmental processes in regions like the cortex, hippocampus, and amygdala [232,233]. Disruptions in ECS signaling during early life, such as exposure to exogenous cannabinoids, can impair neuronal connectivity and lead to long-lasting effects on motor function and seizure susceptibility [234]. The ECS also supports the maturation of stress responses and emotional behaviors during adolescence, contributing to proper neurodevelopmental trajectories [235].

Current preclinical and clinical data indicate that only purified CBD shows consistent efficacy in treating DRE and related neuropsychiatric comorbidities in children. However, given CBD’s potential to modulate the ECS, further studies are necessary to clarify its mechanisms of action and developmental impact. Notably, the long-term effects of chronic CBD exposure on the ECS and brain maturation remain insufficiently understood, highlighting the urgent need for extended follow-up in both clinical and animal studies to determine safe dosing, treatment duration, and the developmental stages at which its use is both effective and safe. Future studies should employ appropriate experimental models focusing on pure CBD and its synthetic derivatives to establish safe and effective dosages, define precise therapeutic targets, and identify analogs that preserve therapeutic benefits while minimizing risks to the developing brain [230].

A different therapeutic approach, supported by preclinical studies, suggests that CB1R ago-PAMs are promising, as they enhance endogenous CB1R signaling preferentially via AEA over 2-AG [236], in an activity-dependent manner, reducing seizure burden in DS and CAE without overstimulation. In contrast, broad CB1R agonism (e.g., THC, WIN552122) often results in mixed or adverse effects, particularly in CAE, where CB1R activation prolongs SWD duration and facilitates seizure generalization. Additionally, FAAH and MAGL inhibitors or dual FAAH MAGL inhibitors, which elevate AEA, 2AG, or both, may restore ECS tone without direct CB1R activation [187,237,238], offering another potential therapeutic strategy for pediatric patients with DRE and CAE.

## 6. Conclusions

In conclusion, alterations in the ECS are likely involved in the pathophysiology of childhood epilepsy. While therapeutic modulation of the ECS holds promise, it is inherently complex and must be approached with caution, as both inhibition and overactivation can cause adverse effects, especially during critical periods of brain development. A deeper understanding of the physiological roles of the ECS is essential to anticipate the consequences of its modulation in pediatric patients and to develop safer therapeutic strategies. Precision targeting of ECS components, considering regional CB1R density, fluctuating eCB levels, and syndrome-specific ECS pathophysiology, may offer a more rational and safer strategy for pediatric epilepsy cases with multifactorial etiologies. This approach may optimize seizure control and address neuropsychiatric comorbidities, particularly in syndromes such as DS, LGS, other DRE, and potentially well-defined subgroups of CAE, but it requires deep mechanistic insights and individualized profiling to avoid therapeutic missteps. However, given the roles of eCBs in development, the long-term use in children warrants caution and further investigation.

## Figures and Tables

**Table 1:** Updated seizure classification based on [16,34].

Seizure Classes	Seizure Types
Focal (F) Seizures	Focal-to-bilateral tonic–clonic (FBTC) seizures Focal preserved consciousness (FPC) seizures Focal impaired consciousness (FIC) seizures Focal epilepsy, temporal lobe epilepsy (TLE), Dravet syndrome
Generalized (G) Seizures	Generalized tonic–clonic seizures Absence seizures, other generalized seizures Absence seizure (AS) Typical absence seizure (TA) Atypical absence seizure (AA) Myoclonic absence seizure (MA) Eyelid myoclonia with/without absence (EMA) Generalized tonic–clonic (GTC) seizure Myoclonic tonic–clonic seizure Absence-to-tonic–clonic seizure Generalized myoclonic (GM) seizure Generalized clonic (GC) seizure Generalized negative myoclonic (GNM) seizure Generalized epileptic spasms (GESs) Generalized tonic (GT) seizure Generalized atonic (GA) seizure Generalized myoclonic–atonic (GMA) seizure
Unknown (U) Seizures	Focal or generalized–preserved consciousness (PC) seizure Focal or generalized–impaired consciousness (IC) seizure Focal or generalized–bilateral tonic–clonic (BTC) seizure Refractory status epilepsy, febrile infection-related epilepsy syndrome (FIRES)
Unclassified	

**Table 2:** Epilepsy syndromes with onset in childhood [35].

Category	Syndromes
Self-Limited Focal Epilepsies	- Self-limited epilepsy with centrotemporal spikes
	- Self-limited epilepsy with autonomic seizures
	- Childhood occipital visual epilepsy
Generalized Epilepsies	- Photosensitive occipital lobe epilepsy
	- Childhood absence epilepsy
Developmental and/or Epileptic Encephalopathies	- Epilepsy with myoclonic absence
	- Epilepsy with eyelid myoclonia
	- Epilepsy with myoclonic–atonic seizures
	- Lennox–Gastaut syndrome
	- Developmental and/or epileptic encephalopathy with spike-and-wave activation in sleep
	- Hemiconvulsion–hemiplegia–epilepsy syndrome

**Table 3:** Summary of the preclinical pharmacological evidence on the effects of cannabinoids in animal models of absence epilepsy.

Model	Cannabinoid	Dose	ROA	Primary Finding	Reference
Sprague Dawley Rats—Photically Evoked After-Discharge Potentials	∆^9^-THC	5 mg/kg	i.p.	↑ Seizure Incidence	[195]
GAERS		0.3, 1, 3, 10 mg/kg 10 mg/kg = ~80 ng/mL ∆^9^-THC plasma concentration	i.p.	↑ Seizure Incidence (200% of Baseline); ↑ Total Time Spent in Seizures; ↑ Seizure Length; ↓ Seizure Frequency	[196]
GAERS		∆^9^-THC ~3 ng/mL plasma concentration	Smoke high-THC cannabis (Mohawk)	↑ Seizure Incidence (>50% Increase); ↑ Total Time Spent in Seizures; ↑ Seizure Length (>100% Increase); ↓ Seizure Frequency	[196]
Sprague Dawley rats—Photically Evoked After-Discharge Potentials	CBD	50 mg/kg	i.p.	↨ Seizure Incidence	[195]
GAERS		30–100 mg/kg 100 mg = ~4000 ng/mL CBD plasma concentration	i.p.	↓ Seizure Incidence (50% Reduction)	[196]
GAERS		~20 ng/mL CBD plasma concentration	Smoke high-CBD cannabis (Treasure Island)	↨ Seizure Incidence	[196]
C57BL/6N Mice	CB1/2R agonist CP55940	0.3 mg/kg	i.p.	↑ High Voltage Spindles Incidence (HVSs)	[197]
WAG/Rij	CB1/2R agonist WIN 55,212-2	3–6–12 mg/kg ED50 value = 4.9 mg/kg	s.c.	First 2 h ↓ Seizure Incidence (80% Reduction after 12 mg/kg) Last 2 h: ↑ Seizure Length (>100 s) ↓ Motor Activity	[198]
WAG/Rij		6 mg/kg	s.c.	↨ Seizures Incidence in the First 3 h and in 24 h Recording; ↑ Seizure Length (>11 s) ↨ Motor Activity	[199]
WAG/R		6 mg/kg (subchronic = 3 times a week for 2 weeks)	s.c.	↑ Seizure Length (>11 s) ↨ Motor Activity	[199]
WAG/R		0.1–0.3–1–2 μg/2 μL	i.c.v.	↓ Seizure Incidence ↓ Seizure Total Time	[200]
WAG/R		0.1–0.3–1 μL/0.5 μL	NRT/VB/S1po Bilateral Brain Infusion	↓ Seizure Incidence ↓ Seizure Total Time	[200]
WAG/Rij	CB1R antagonist AM251	6–12 mg/kg	s.c.	↨ Seizures Incidence	[198]
C57BL/6N Mice	CP55940 + AM251	0.3 mg/kg = 3 mg/kg	i.p.	↨ High Voltage Spindles Incidence (HVSs)	[197]
WAG/Rij	WIN 55,212-2 +AM251	WIN 6 mg/kg + AM 12 mg	s.c.	↨ Seizures Incidence in the First 3 h ↑ Seizure Incidence in 4th and 5th h ↨ Last 2 h Seizure Length	[198]
GAERS (♂)	GAT211 (Ago-PAM)	3, 10 mg/kg	i.p.	↓ Seizure Incidence ↓ Total Time Spent in Seizures (~40%); ↨ Seizure Length ↨ Seizure Frequency	[201]
GAERS (♂♀)	GAT229 (AGO-PAM)	1, 3, 10 mg/kg	i.p.	↓ Seizure Incidence ↓ Total Time Spent in Seizures (~40%); ↨ Seizure Length ↨ Seizure Frequency ↨ Motor Activity (Open-Field Test)	[201]
GAERS (♂)		125, 250, 500, 1000 μM	Cortical (motor Cx) infusion	↓ Seizure Incidence ↓ Total Time Spent in Seizures ↨ Seizure length ↨ Seizure Frequency	[201]
GAERS (♂)	SR141716A (CB1R antagonist)	3 mg/kg	i.p.	↨ Seizure Incidence ↨ Total Time Spent in Seizures	[201]
WAG/Rij		0.5, 1, and 2 μg/2 μL	i.c.v.	↑ Seizure Incidence ↑ Total Time Spent in Seizures (>40% Increase);	[202]
WAG/Rij		0.5–1–2.5 μg/0.5 μL	VB Bilateral Brain Infusion	↑ Seizure Incidence (>50% Increase); ↑ Total Time Spent in Seizures	[200]
WAG/Rij		0.5–1–2.5 μg/0.5 μL	NRT/S1po Bilateral Brain Infusion	↨ Seizure Incidence (>50% Increase); ↨ Total Time Spent in Seizures	[200]
GAERS (♂)	GAT229 + SR141716A	1000 μM 3 mg/kg	Cortical infusion i.p.	↨ Seizure Incidence ↨ Total Time Spent in Seizures	[201]
WAG/Rij	*N*-palmitoylethanolamine (PEA) + SR141716	40 mg/kg + 0.5 μg/2 μL	i.p. + i.c.v.	↨ Seizure Incidence ↨ Total Time Spent in Seizures	[202]
WAG/Rij	GW6471 (PPAR-α antagonist)	1, 2 μg/2 μL	i.c.v.	↨ Seizure Incidence ↨ Total Time Spent in Seizures	[202]
WAG/Rij	PEA + GW6471	3 μg/2 μL + 2 μg/2 μL	i.c.v. + i.c.v.	↨ Seizure Incidence ↨ Total Time Spent in Seizures	[202]
GAERS (♂)	GAT591 (AGO-PAM)	1, 3, 10 mg/kg	i.p.	↨ Seizure Incidence ↓ Total Time Spent in Seizures ↨ Seizure Length Seizure Frequency	[203]
GAERS (♂)	GAT593 (AGO-PAM)	1, 3, 10 mg/kg	i.p.	↨ Seizure Incidence ↓ Total Time Spent in Seizures ↨ Seizure Length ↨ Seizure Frequency	[203]
WAG/Rij	PEA	0.5, 1,3 and 10 μg/2 μL	i.c.v.	↑ Seizure Incidence ↓ Total Time Spent in Seizures	[202]
		10, 20, 40, 60 mg/kg	i.p.		
WAG/Rij	anandamide (*N*-arachidonylethanolamine, AEA)	1, 3 and 10 μg/2 μL	i.c.v.	↑ Seizure Incidence ↓ Total Time Spent in Seizures	[202]
WAG/Rij	AEA + SR141716	3 μg/2 μL + 0.5 μg/2 μL	i.c.v. + i.c.v.	↨ Seizure Incidence ↨ Total Time Spent in Seizures	[202]
WAG/Rij	AEA + GW6471	3 μg/2 μL + 2 μg/2 μL	i.c.v. + i.c.v.	↑ Seizure Incidence ↓ Total Time Spent in Seizures	[202]

The table includes various cannabinoids, their respective doses, routes of administration (ROAs), the animal models used, and the primary findings related to seizure activity. The abbreviations used in this table are as follows: ∆9-THC refers to delta-9-tetrahydrocannabinol, CBD stands for cannabidiol, and CB1R/CB2R represent cannabinoid receptors 1 and 2. The GAERS model refers to Genetic Absence Epilepsy Rats from Strasbourg, and WAG/Rij indicates the Wistar Albino Glaxo/Rijswijk rat strain. The term C57BL/6N mice refers to a commonly used inbred strain of mice. Routes of administration are indicated as i.p. (intraperitoneal injection), s.c. (subcutaneous injection), and i.c.v. (intracerebroventricular injection). The table also uses NRT/VB/S1po to refer to the nucleus reticularis thalami, ventrobasal thalamus, and S1 posterior, which are part of the thalamocortical pathway. Other terms include Ago-PAM (agonist-positive allosteric modulator), PPAR-α (peroxisome proliferator-activated receptor alpha), SR141716A (a CB1 receptor antagonist), PEA (N-palmitoylethanolamine), and AEA (anandamide). ↑ increase, ↓ decrease, and ↨ no change.
